# Phenotypic and Genotypic Analyses of Antimicrobial Resistance Patterns of *Staphylococcus aureus* Isolates From Outpatient Blood Samples in Mukuru Slum, Nairobi, Kenya

**DOI:** 10.1155/cjid/3974296

**Published:** 2026-04-28

**Authors:** Jeremiah Ogeto, Simon Mitema, Gabriel Aboge, Alfred Mainga, Gervason Moriasi

**Affiliations:** ^1^ Department of Public Health Pharmacology and Toxicology, Faculty of Veterinary Medicine, University of Nairobi, PO Box 29053-00625, Nairobi, Kenya, uonbi.ac.ke; ^2^ Department of Biochemistry, Microbiology and Biotechnology, Kenyatta University, PO BOX 43844-00100-GPO, Nairobi, Kenya, ku.ac.ke; ^3^ Department of Medical Biochemistry, Mount Kenya University, PO BOX 342-01000, Thika, Kenya, mku.ac.ke

**Keywords:** antimicrobial resistance, bloodstream infection, Kenya, MRSA, Mukuru slum, Nairobi, *Staphylococcus aureus*

## Abstract

**Background:**

Bloodstream infections caused by *Staphylococcus aureus* (*S. aureus*) and its methicillin‐resistant *S. aureus* (MRSA) represent a major clinical challenge, contributing to prolonged illness, increased healthcare costs, and high mortality worldwide. In low‐resource settings, these infections are further aggravated by overcrowding, poor sanitation, and limited access to healthcare. Mukuru, the second‐largest informal settlement in Nairobi, Kenya, is characterized by such socioenvironmental conditions, which plausibly facilitate the transmission and persistence of antimicrobial‐resistant pathogens, including MRSA. Therefore, the aim of the study was to characterize the phenotypic and genotypic antibiotic resistance patterns of *S. aureus* isolated from outpatient blood samples collected in Mukuru Slum settlement.

**Methods:**

This cross‐sectional study analyzed blood samples collected from outpatients in Mukuru informal settlement. *S. aureus* isolates were identified using standard microbiological cultures and biochemical tests. Antimicrobial susceptibility testing (AST) was performed using the Kirby–Bauer disk diffusion method against a panel of 10 antibiotics. The molecular detection of antimicrobial resistance genes (*mecA*, *mecC*, *gyrA*, *gyrB*, and *tetM*) was conducted using polymerase chain reaction (PCR), agarose gel electrophoresis, and sequencing (*mecA*).

**Results:**

A total of 53 *S. aureus* isolates were recovered. Phenotypic resistance rates were sulfamethoxazole–trimethoprim (54.7%), ampicillin (50.9%), cefoxitin (37.7%), tetracycline (35.9%), ciprofloxacin (28.3%), gentamicin (28.3%), amoxicillin–clavulanic acid (28.3%), and erythromycin (18.9%). Multidrug resistance (MDR) was observed in 35.8% of the isolates. Genotypic analysis revealed that 82.4% of the cefoxitin‐resistant MRSA isolates harbored the *mecA* gene, while *mecC* was not detected. Among ciprofloxacin‐resistant isolates, 91.7% possessed the *gyrA* gene and 75.0% carried *gyrB*. The *tetM* gene was detected in 60.0% of tetracycline‐resistant isolates.

**Conclusion:**

The study reveals a high prevalence of MDR *S. aureus* and MRSA in the study population, driven by significant resistance to commonly prescribed antibiotics such as penicillin and trimethoprim–sulfamethoxazole. The strong concordance between phenotypic resistance and the presence of *mecA*, *gyrA*, and *tetM* genes underscores the genetic basis of resistance in this setting. These findings highlight the urgent need for enhanced antimicrobial stewardship and infection control measures in informal settlements.

## 1. Introduction

*Staphylococcus aureus* is among the most notorious causes of hospital‐ and community‐acquired invasive and soft tissue infections, with nosocomial infections by methicillin‐resistant *S. aureus* (MRSA) now reported worldwide [1]. MRSA strains are increasingly resistant to multiple antimicrobial classes, including β‐lactams, fluoroquinolones, and tetracyclines, posing a significant challenge to global public health [2]. Of particular concern is the rising resistance to vancomycin and newer glycopeptides, which are often considered last‐resort treatments for MRSA infections [3].

Methicillin resistance in staphylococci is primarily mediated by the *mecA* or *mecC* genes, located on the Staphylococcal Cassette Chromosome *mec* (*SCCmec*) [4]. These genes encode penicillin‐binding protein 2A (PBP2A), which has a reduced affinity for β‐lactam antibiotics and carbapenems, rendering them ineffective [5]. However, newer cephalosporins such as ceftaroline and ceftobiprole retain activity against MRSA due to their unique binding properties [6]. The *gyrA* and *gyrB* genes, encoding subunits of DNA *gyrA*se, are critical targets of fluoroquinolones, and their mutations can lead to high‐level resistance, further limiting treatment options [7]. Additionally, the *tetM* gene, frequently found on transposons and plasmids, confers resistance to tetracyclines, a class of antibiotics historically used to treat MRSA infections [8].

The colocalization of *tetM* with *SCCmec* on mobile genetic elements facilitates the horizontal transfer of resistance genes, promoting the rapid dissemination of MDRSA strains [5]. This genetic plasticity, combined with the selective pressure exerted by widespread antibiotic use, has led to the global proliferation of MRSA strains resistant to multiple drug classes, thereby complicating therapeutic interventions in human and veterinary medicines [9].

MRSA can be categorized into three groups: hospital‐acquired MRSA (HA‐MRSA), community‐acquired MRSA (CA‐MRSA), and livestock‐associated MRSA (LA‐MRSA) [10]. Typically, HA‐MRSA strains possess *SCCmec* I, II, or III, and based on multilocus sequencing, *SCCmec* I harbors the *mecA* gene as the sole dominant for resistance to β‐lactams. At the same time, *SCCmec* II and *SCCmec* III have several resistance determinants to non‐β‐lactam antibiotics, demonstrating multidrug resistance (MDR) of nosocomial MRSA isolates [11]. CA‐MRSA strains have a wide distribution of *SCCmec* element types IV, V, and VII and appear to harbor exotoxin Panton‐Valentine Leucocidin (PVL), which proportionately determines the severity of infection [12]. Besides, CA‐MRSA cause potentially fatal illnesses mediated by the PVL toxin, which causes necrotizing fasciitis, septic thrombophlebitis, septic arthritis, bacteremia, post‐influenza pneumonia, and hemorrhagic pneumonia [6]. LA‐MRSA colonizes animals such as pigs or calves and results in human diseases upon contact with infected animals or their products, especially meat and milk [6], demonstrating the zoonotic nature of MRSA strains [13].

The median prevalence of MRSA infections in humans in the African Mediterranean region is about 39%; however, higher prevalence rates (50%) have been reported in Malta, Cyprus, Egypt, and Jordan [14]. Besides, MRSA prevalence in East African countries ranges from 31% to 82% in Uganda and Rwanda, 10%–50% in Tanzania, and 3.5%–53.4% in Kenya [15]. However, there is a paucity of evidence‐based data on the prevalence and resistance patterns of MDRSA and MRSA strains in sub‐Saharan African counties like Kenya, especially in its informal urban settlements to guide therapeutic and infection control interventions and antimicrobial resistance stewardship programs. Therefore, this study phenotypically and genotypically characterized the MDRSA, including MRSA, in blood samples drawn from patients receiving outpatient services in hospitals within the Mukuru slum in Nairobi County.

## 2. Materials and Methods

### 2.1. Study Area

**FIGURE 1:**
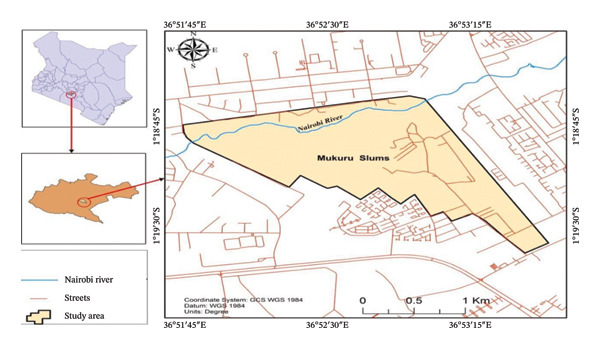
Map of Kenya showing Mukuru Kwa Njenga slum (adapted from Kaburu et al. [16]).

The study was conducted on clinical blood specimens obtained from outpatients attending healthcare facilities within the Mukuru informal settlement, Nairobi City County, Kenya (Figure 1), between July and November 2024. Mukuru is an informal settlement located approximately 5–7 km from the Nairobi central business district, and the second‐largest informal settlement in Kenya, encompassing the clusters of Viwandani, Mukuru Kwa Reuben, and Mukuru Kwa Njenga. It spans approximately 525 acres and, according to the Kenya National Bureau of Statistics, contains about 100,561 households with an estimated population of over 700,000 residents [17]. Geographically, Mukuru is situated at approximately 1.3167°S latitude and 36.8667°E longitude, on the southeastern periphery of Nairobi city’s industrial area. The settlement is characterized by extremely high population density and persistent infrastructural deficits, including inadequate access to clean water, sanitation facilities, waste management, roads, drainage, and electricity. These socioenvironmental challenges contribute to a high burden of communicable diseases such as diarrheal illness, upper respiratory tract infections, malaria, and febrile syndromes [18]. Healthcare services within Mukuru are primarily delivered by a mix of outpatient community health centers, mission‐run clinics, and small private outpatient facilities that provide basic curative services but have limited diagnostic capacity and restricted access to advanced laboratory testing. Such conditions not only intensify the risk of disease transmission but also create an ecological niche that favors the emergence and spread of antimicrobial‐resistant pathogens [18]. In particular, the combination of overcrowding, frequent infectious disease episodes, and limited healthcare access increases the likelihood of indiscriminate antibiotic use and suboptimal treatment. This made Mukuru a critical setting for investigating the prevalence, resistance patterns, and molecular characteristics of *S. aureus* and MRSA, with implications for both local disease control and broader public health surveillance.

### 2.2. Blood Sample Collection

Consenting patients with a history of fever lasting 3 days or more, an axillary temperature of ≥ 37.5°C, and ≥ 3–8 diarrheal episodes with visible blood within 24 h prior were conveniently selected for blood sampling. The blood samples were obtained aseptically through the venipuncture technique by qualified medical practitioners, labeled using unique reference numbers, packaged individually in Ziplock bags, and then transported in a cooler box to our laboratory for bacteriological analysis.

### 2.3. Isolation and Identification of *S. aureus*

Blood samples were collected aseptically and inoculated into Tryptone Soya Broth (TSB) (Oxoid Ltd., Basingstoke, UK). The inoculated TSB was incubated at 37°C for 3 days to allow for bacterial growth. Turbid broths were subcultured onto Mannitol Salt Agar (MSA) (Becton, Dickinson and Company, Sparks, MD, USA) and incubated at 37°C for 24–48 h. Characteristic yellow colonies on MSA were purified on nutrient agar and identified as *S. aureus* based on colony morphology, Gram staining characteristics, and biochemical tests including catalase and coagulase tests, β‐hemolysis on sheep blood agar, and mannitol fermentation following standard microbiological identification procedures.

Genomic DNA was extracted from overnight *S. aureus* colonies using a boiling cell lysis method, which was employed specifically for *nuc* gene PCR‐based confirmation of *S. aureus* identity given its simplicity and suitability for screening a large number of isolates. Briefly, 2–3 colonies were suspended in 200 μL of sterile nuclease‐free water, heated at 95°C for 10 min to lyse the cells, and immediately cooled on ice. The lysates were centrifuged at 12, 000 × *g* for 5 min, and the supernatant containing crude genomic DNA was collected. DNA yield was assessed using a NanoDrop 2000 (Thermo Scientific, Wilmington, DE, USA), and aliquots were stored at −20°C until *nuc* gene PCR analysis [19].

Conventional polymerase chain reaction (PCR) was performed to amplify the *nuc* gene (specific for *S. aureus*) using the following primer set: forward 5′‐GCG​ATT​GAT​GGT​GAT​ACG​GTT‐3′ and reverse 5′‐CAA​GCC​TTG​ACG​AAC​TAA​AGC‐3′ [20]. Each 25‐μL PCR reaction contained 12.5 μL of 2× PCR Master Mix (Promega), 0.5 μM of each primer, 2 μL of DNA template, and nuclease‐free water to volume. Amplification was carried out in a thermocycler with the following cycling conditions: initial denaturation at 95°C for 5 min; 35 cycles of denaturation at 95°C for 30 s, annealing at 55°C for 30 s, and extension at 72°C for 1 min; followed by a final extension at 72°C for 5 min. After that, the PCR products were separated on 1.5% (w/v) agarose gels stained with ethidium bromide, run with a 100‐bp Promega molecular ladder at 100 V for 45 min, and visualized under UV light using a GelMax 125 Imager (Cambridge, UK) with UVP software (Upland, CA, USA).

### 2.4. Antimicrobial Susceptibility Testing

Antimicrobial susceptibility testing was performed using the Kirby–Bauer disk diffusion method in accordance with the Clinical and Laboratory Standards Institute (CLSI) guidelines [21]. A panel of 10 antibiotic disks (Oxoid Ltd., Basingstoke, UK) was used: cefoxitin (FOX, 30 μg), benzylpenicillin (PEN, 10 units), ciprofloxacin (CIP, 5 μg), erythromycin (ERY, 15 μg), gentamicin (CN, 10 μg), tetracycline (TET, 30 μg), amoxicillin–clavulanic acid (AMC, 20/10 μg), trimethoprim–sulfamethoxazole (SXT, 1.25/23.75 μg), chloramphenicol (C, 30 μg), and clindamycin (DA, 2 μg). The disk‐impregnated plates (in triplicate) were incubated at 37°C for 24 h, after which the growth inhibition zones were measured using a Vernier caliper. The zones of inhibition were interpreted as susceptible, intermediate, or resistant according to CLSI breakpoints (Table 1). *S. aureus* isolates resistant to at least one antibiotic in three or more antibiotic classes were classified as MDR, while isolates exhibiting an inhibition zone diameter of ≤ 21 mm to cefoxitin were considered MRSA as per CLSI guidelines [22, 23]. *S. aureus* ATCC 25923 was used for quality control, while distilled water (DH_2_O) was used as a negative control [21].

**TABLE 1:** Interpretive category and zone diameter breakpoints for antibiotic susceptibility testing.

Antibiotic agent	Concentration (µg)	Zone diameter breakpoints (mm)		
		Susceptible (S)	Intermediate (I)	Resistant (R)
Cefoxitin	30	≥ 22	—	≤ 21
Ampicillin	10	≥ 17	—	≤ 16
Ciprofloxacin	5	≥ 21	16–20	≤ 15
Erythromycin	15	≥ 23	14–22	≤ 13
Gentamicin	10	≥ 15	13–14	≤ 12
Tetracycline	30	≥ 19	15–18	≤ 14
Amoxicillin/Clavulanic Acid	30	≥ 20	—	≤ 19
Sulfamethoxazole/Trimethoprim	25	≥ 16	11–15	≤ 10

*Note:* Adapted from the CLSI 2017 guideline [21].

### 2.5. Molecular Characterization of Antimicrobial Resistance Genes

Genomic DNA was extracted from overnight bacterial cultures using a commercial DNA extraction kit (QIAamp DNA Mini Kit, Qiagen, Hilden, Germany) following the manufacturer’s instructions. This kit‐based extraction method was employed for antimicrobial resistance gene screening, as it yields higher‐purity DNA than the boiling cell lysis method used in Section 2.3 for *nuc* gene confirmation; reliable amplification of *mecA*, *mecC*, *gyrA*, *gyrB*, and *tetM* requires a more stringent extraction protocol to ensure consistent PCR performance. DNA concentration and purity were assessed using A260/280 absorbance ratios on a NanoDrop 2000 (Thermo Scientific, Wilmington, DE, USA), and aliquots were stored at −20°C until use. PCR was performed to detect the presence of resistance genes (*mecA*, *mecC*, *gyrA*, *gyrB*, and *tetM*) using specific primers (Inqaba Biotech, South Africa). The PCR reaction mixture (total volume 25 μL) contained 12.5 μL of Master Mix (GoTaq® Green Master Mix, Promega, Madison, WI, USA), 0.5 μM of each primer (forward and reverse), 2.0 μL of template DNA, and 8.5 μL of nuclease‐free water. The primer sequences and expected amplicon sizes are detailed in Table 2. PCR cycling conditions were as follows: initial denaturation at 95°C for 5 min, followed by 35 cycles of denaturation at 95°C for 30 s, annealing at 50–60°C (variable per gene; Table 2) for 30 s, and extension at 72°C for 1 min, with a final extension at 72°C for 10 min. Amplicons and a molecular ladder (100 bp, Promega) were separated through agarose gel electrophoresis (AGE) on a 1.5% agarose gel stained with ethidium bromide dye. The bands were visualized using a UV trans‐illuminator digital camera (Gelmax 125 imager, Cambridge, UK) connected to a computer running UVP software (UVP, Upland, CA, USA).

**TABLE 2:** Target antimicrobial resistance–associated genes and their specific primers.

Primer	Oligonucleotide sequence (5′–3′)	Annealing temperature (°C)	Target gene	Amplicon size (bp)	Reference
mecA(F)	AAA​ATC​GAT​GGT​AAA​GGT​TGG​C	53	*mec*A	533	[24]
mecA(R)	AGT​TCT​GGA​GTA​CCG​GAT​TTG​C				

mecC‐F	GAA​AAA​AAG​GCT​TAG​AAC​GCC​TC	59	*mec*C	138	[25]
mecC‐R	GAA​GAT​CTT​TTC​CGT​TTT​CAG​C				

gyrA(F)	ACGCAAGAGAGATGGTT	45	*gyr*A	270	[26]
gyrA(R)	TCAGTATAACGC ATCGC AGC				

gyrB(F)	ATG​GCA​GCT​AGA​GGA​AGA​GA	53	*gyr*B	382	[26]
gyrB(R)	GTGATCCATCA ACATCC GCA				

tetM (F)	GAG​GTC​CGT​CTG​AAC​TTT​GCG	56	*tet*M	580	[27]
tetM (R)	AGA​AAG​GAT​TTG​GCG​GCA​CT				

nuc‐ (F)	GCG​ATT​GAT​GGT​GAT​ACG​GTT	50	*nuc*	276	[28]
nuc‐(R)	CAA​GCC​TTG​ACG​AAC​TAA​AGC				

### 2.6. Sequencing and Sequence Analysis

PCR amplicons of the *nuc* and *mecA* genes generated from 10 selected *S. aureus* isolates were purified prior to sequencing. Amplicon purification was performed using the QIAquick PCR Purification Kit (Qiagen, Hilden, Germany) according to the manufacturer’s protocol to remove unincorporated primers, nucleotides, and other PCR reaction components. The purified products were shipped under cold chain to Macrogen Europe B.V. (Amsterdam, The Netherlands) for bidirectional Sanger sequencing using the same primers employed in PCR amplification.

The raw chromatogram files (.ab1) were quality‐checked using Chromas Lite v2.6.6 (Technelysium Pty Ltd, Australia). Low‐quality bases at both ends were trimmed, and forward and reverse reads were assembled into consensus sequences using BioEdit v7.2.6. The final consensus sequences were queried against the nonredundant nucleotide database at the National Center for Biotechnology Information (NCBI) using the Basic Local Alignment Search Tool (BLASTN). Percentage identity, query coverage, and *E*‐values were recorded to determine the similarity of study isolates with reference sequences. Multiple sequence alignments were generated using ClustalW implemented in MEGA X software to assess the intrastudy sequence variation. Representative sequences were submitted to GenBank through the NCBI BankIt submission portal, and accession numbers were obtained for public archiving and referencing in this manuscript.

### 2.7. Data Management and Analysis

Phenotypic and genotypic data for *S. aureus* resistance were tabulated in an MS Excel spreadsheet. A descriptive analysis of the data was performed using GraphPad Prism software (Version 10.3), and the results were presented in tables. Qualitative data from AGE were visualized, and the electropherograms were photographed, presented, and described.

### 2.8. Ethical Considerations

Ethical approval was sought and obtained from the University of Nairobi’s Faculty of Veterinary Medicine’s Biosecurity, Animal Use and Ethics Committee (REF: FVM BAUEC/2024/301). Besides, written informed consent was obtained from all participants or their legal guardians prior to their involvement in the study. The confidentiality of patient information was rigorously maintained at all stages to ensure the privacy and security. All study procedures were conducted in strict accordance with the ethical principles outlined in the Declaration of Helsinki.

## 3. Results

### 3.1. Isolation and Identification of *S. aureus*

Of the 142 blood samples processed, 53 (37.3%) were confirmed as *S. aureus* based on standard cultural and biochemical characteristics, including β‐hemolysis (112/142; 78.9%), coagulase positivity (56/142; 39.4%), and mannitol fermentation on MSA (Supporting Figure 1 (SF. 1; Supporting Table 1 (ST. 1)), and further confirmed molecularly by PCR amplification of the *nuc* gene (276 bp) in 10 selected isolates (Supporting Figure 2 (SF. 1). Colony morphologies on MSA are presented in Supporting File 1. BLASTn analysis of the *nuc* gene amplicons from these 10 sequenced isolates confirmed *S. aureus* identity, with nucleotide identities ranging from 96% to 100% (Table 3).

**TABLE 3:** Nuclease genes of 10 selected *S. aureus* isolates and their sequenced homologue and identity obtained from NCBI GenBank using nucleotide–nucleotide BLASTn.

Isolate ID	Target gene	Homologue	*E*‐value	Nucleotide % identity	Accession number
80 SAR	*nuc* gene	*S. aureus*	2*e* − 116	99	OL614664
115 SAR	*nuc* gene	*S. aureus*	3*e* − 120	99	OL614665
469 SAF	*nuc* gene	*S. aureus*	2*e* − 117	100	OL614666
471 SAR	*nuc gene*	*S. aureus*	2*e* − 117	100	OL614667
476 SAR	*nuc* gene	*S. aureus*	3*e* − 120	99	OL614669
479 SAF	*nuc* gene	*S. aureus*	7*e* − 118	99	OL614670
484 SAF	*nuc* gene	*S. aureus*	2*e* − 117	100	OL614672
487 SAR	*nuc* gene	*S. aureus*	2*e* − 108	96	OL614673
491 SAR	*nuc* gene	*S. aureus*	5*e* − 118	100	OL614674
493 SAF	*nuc* gene	*S. aureus*	5*e* − 116	99	OL614675

Sanger sequencing of *nuc* gene PCR amplicons from the 10 selected isolates (80SAR, 115SAR, 469SAF, 471SAR, 476SAR, 479SAF, 484SAF, 487SAR, 491SAR, and 493SAF) confirmed *S. aureus* identity, with nucleotide identities of 96% (487SAR), 99% (80SAR, 115SAR, 476SAR, 479SAF, 493SAF), and 100% (469SAF, 471SAR, 484SAF, 491SAR). GenBank accession numbers were obtained for all 10 sequenced isolates (Table 3).

### 3.2. Antimicrobial Susceptibility of the *S. aureus* Isolates

Antimicrobial susceptibility testing of all 53 confirmed *S. aureus* isolates revealed that 50 (94.34%) exhibited resistance to at least one of the eight primary antimicrobial agents included in the MDR phenotypic classification panel (Table 4; Supporting Table 2). Three isolates did not exhibit resistance to any of the eight antimicrobial agents constituting the primary classification panel (cefoxitin, ampicillin, ciprofloxacin, erythromycin, gentamicin, tetracycline, amoxicillin–clavulanic acid, sulfamethoxazole–trimethoprim) and are therefore not represented in Tables 4 or 5. The highest resistance was observed for sulfamethoxazole–trimethoprim (29/53; 54.7%) and ampicillin (27/53; 50.9%) (Table 4; Supporting Table 2). Moderate resistance was detected for cefoxitin (20/53; 37.7%) and tetracycline (19/53; 35.9%) (Table 4; Supporting Table 2). Lower resistance frequencies were noted for ciprofloxacin (15/53; 28.3%), erythromycin (10/53; 18.9%), gentamicin (15/53; 28.3%), and amoxicillin–clavulanic acid (15/53; 28.3%) (Table 4; Supporting Table 2).

**TABLE 4:** Susceptibility of the *S. aureus* isolates obtained from human blood to various antimicrobial agents.

Antibiotics	AST (*n* = 53)	
	R (%)	S (%)
Cefoxitin (30 μg)	20 (37.74)	33 (62.26)
Ampicillin (10 μg)	27 (50.94)	26 (49.06)
Ciprofloxacin (5 μg)	15 (28.30)	38 (71.70)
Erythromycin (15 μg)	10 (18.87)	34 (64.15)
Gentamicin (10 μg)	15 (28.30)	37 (69.81)
Tetracycline (30 μg)	19 (35.85)	34 (64.15)
Amoxicillin/Clavulanic acid (30 μg)	15 (28.30)	38 (71.70)
Sulfamethoxazole/Trimethoprim (25 μg)	29 (54.74)	22 (41.51)

*Note:* Key: R, resistant; S, susceptible.

**TABLE 5:** Antimicrobial resistance (AMR) and multidrug resistance (MDR) phenotypes of *S. aureus* isolates from human blood.

Antimicrobial‐resistant phenotypes	Proportion (*n* (%)	Resistance phenotype
FOX–AMP–CIP–ERY–GENT–TET–AMC–SXT	4 (7.55)	MDR
FOX–AMP–CIP–ERY–GENT–AMC–SXT	1 (1.88)	MDR
FOX–AMP–CIP–GENT–AMC–SXT	8 (15.09)	MDR
FOX–AMP–CIP–ERY–GENT–AMC	1 (1.88)	MDR
FOX–AMP–CIP–GENT–AMC	1 (1.88)	MDR
FOX–AMP–SXT	1 (1.88)	MDR
AMP–TET–SXT	1 (1.88)	MDR
AMP–ERY–SXT	1 (1.88)	MDR
ERY–TET–SXT	1 (1.88)	MDR
FOX–AMP	2 (3.77)	Non‐MDR
ERY–SXT	2 (3.77)	Non‐MDR
TET–SXT	3 (5.66)	Non‐MDR
AMP–TET	2 (3.77)	Non‐MDR
FOX	2 (3.77)	Non‐MDR
AMP	5 (9.43)	Non‐MDR
SXT	7 (13.20)	Non‐MDR
TET	8 (15.09)	Non‐MDR

*Note:* Key: FOX (cefoxitin), AMP (ampicillin), CIP (ciprofloxacin), ERY (erythromycin), GENT (gentamicin), TET (tetracycline), AMC (amoxicillin–clavulanic acid), SXT (sulfamethoxazole–trimethoprim). MDR, multidrug resistance (nonsusceptibility to at least one agent in three or more antimicrobial classes); non‐MDR, resistance to antimicrobial agents in one or two classes only.

Overall, most isolates remained susceptible to ciprofloxacin, erythromycin, gentamicin, and amoxicillin–clavulanic acid, with susceptibility rates ranging between 64% and 72% (Table 4; Supporting Table 2). Resistance to cefoxitin confirmed the presence of MRSA among 37.7% of the isolates (Table 4; Supporting Table 2). Notably, the majority of isolates (62.3%) were cefoxitin‐susceptible and therefore classified as methicillin‐sensitive *S. aureus* (MSSA) (Table 4; Supporting Table 2).

### 3.3. MDR of *S. aureus* to Various Antimicrobial Agents

In this study, 50 (94.34%) of the 53 confirmed isolates of *S. aureus* obtained from human blood exhibited resistance to at least one antimicrobial agent within the primary classification panel (Table 5), of which 19 (35.85%) met the criteria for MDR as defined in Section 2.4. The remaining 31 isolates were resistant to antibiotics in one or two classes only and are classified as non‐MDR in Table 5. Generally, nine MDR phenotypes were observed among the *S. aureus* isolates obtained from human blood samples, with the most predominant being the FOX–AMP–CIP–GENT–AMC–SXT (15.09%) and FOX–AMP–CIP–ERY–GENT–TET–AMC–SXT (7.55%) (Table 5). In addition, the less frequently detected MDR phenotypes among *S. aureus* isolates from human blood were FOX–AMP–CIP–ERY–GENT–AMC–SXT, FOX–AMP–CIP–ERY–GENT–AMC, FOX–AMP–CIP–GENT–AMC, FOX–AMP–SXT, AMP–TET–SXT, AMP–ERY–SXT, and ERY–TET–SXT with a prevalence of 1.88% (Table 5).

### 3.4. Detection of Antimicrobial Resistance Genes

Molecular screening was performed for *mecA*, *mecC*, *gyrA*, *gyrB*, and *tetM* genes (Figure 2(a)) in targeted subgroups based on phenotypic resistance profiles. Specifically, the *mecA* and *mecC* genes were screened in 17 (85%) of the 20 cefoxitin‐resistant isolates, and three were excluded due to insufficient DNA yield following repeated extraction attempts. The *gyrA* and *gyrB* genes were screened in 12 of the 14 ciprofloxacin‐resistant isolates that fell within the cefoxitin‐resistant screened group (*gyrA*/*gyrB* PCR was inconclusive for two isolates due to template limitations). The remaining ciprofloxacin‐resistant isolate that did not coexhibit cefoxitin resistance (i.e., a methicillin‐susceptible *S. aureus*, MSSA, phenotype) was not screened for *gyrA*/*gyrB* in this study, as the molecular screening strategy was primarily designed to characterize resistance determinants in the context of MRSA coresistance. The absence of *gyrA*/*gyrB* screening in this single MSSA‐CIP isolate represents a limitation of the current study, and future work should assess fluoroquinolone resistance determinants across all ciprofloxacin‐resistant isolates regardless of methicillin resistance status. The *tetM* gene was screened independently in all 19 tetracycline‐resistant isolates, regardless of the cefoxitin resistance status, given that tetracycline resistance is genetically independent of methicillin resistance. Among the 17 phenotypically cefoxitin‐resistant (MRSA) isolates screened for *mecA*, 14 (82.35%) harbored the *mecA* gene (Figure 2(a)), while none harbored the *mecC* gene. The remaining three isolates (17.65%) in this group did not harbor either *mecA* or *mecC*. The *tetM* gene was present in 60.0% (Figure 2(b)) of tetracycline‐resistant isolates. For quinolone resistance, the *gyrA* gene (Figure 2(c)) was detected in 91.7% of ciprofloxacin‐resistant isolates, while *gyrB* (Figure 2(d)) was detected in 75.0% of these isolates.

FIGURE 2(a–d): Ethidium bromide–stained agarose gel electropherograms of PCR‐amplified antimicrobial resistance genes in selected *S. aureus* isolates: (a) mecA gene (533 bp), (b) tetM gene (580 bp), (c) gyrA gene (270 bp), and (d) gyrB gene (382 bp); L: molecular ladder.

**Figure:**
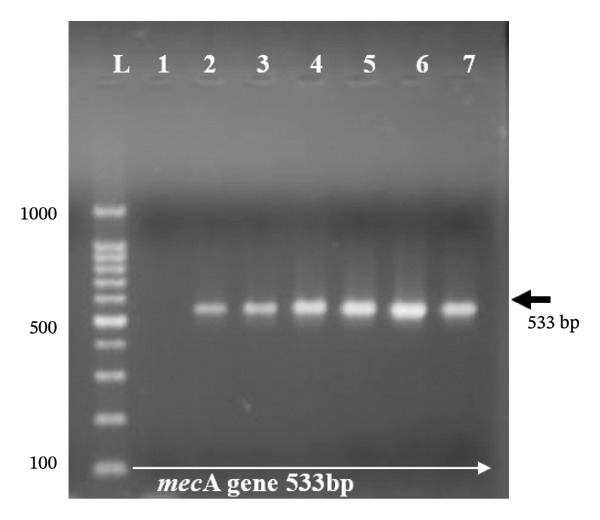
(a)

**Figure:**
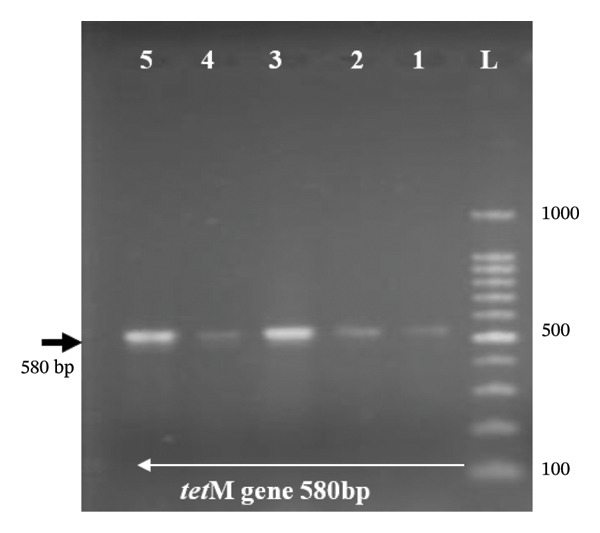
(b)

**Figure:**
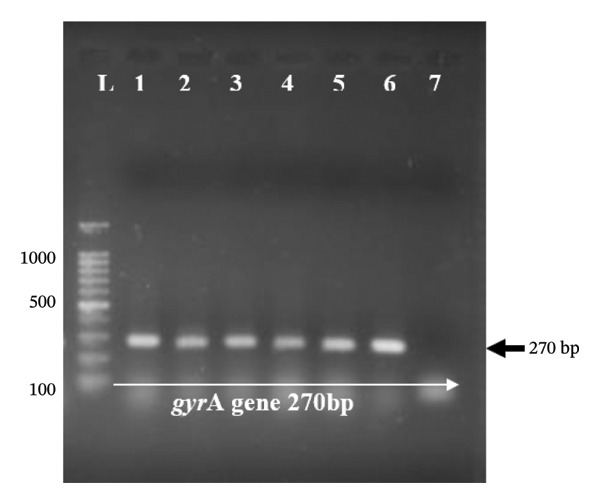
(c)

**Figure:**
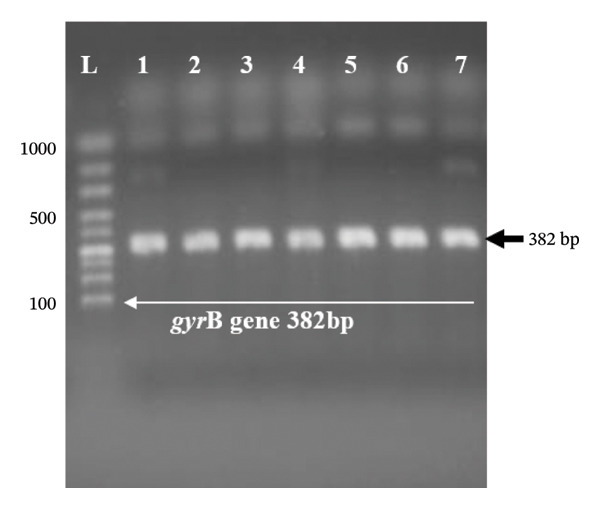
(d)

### 3.5. Genotype–Phenotype Concordance in MDR Isolates

MDR was observed in 19 (35.85%) of the isolates. Various resistance genotypes were detected in each of the 17 molecularly characterized MDR *S. aureus* isolates, with each isolate’s phenotypic resistance profile directly aligned with the detected genetic determinants (Table 6). Of the four isolates exhibiting the FOX–AMP–CIP–ERY–GENT–TET–AMC–SXT phenotype (MDR‐01 to MDR‐04), three (MDR‐01, MDR‐02, MDR‐03) coharbored *mecA*, *gyrA*, *gyrB*, and *tetM*, consistent with their phenotypic resistance to cefoxitin (FOX), ciprofloxacin (CIP), and tetracycline (TET), respectively. In contrast, MDR‐04—exhibiting the same phenotype—harbored only *mecA*, suggesting that its fluoroquinolone and tetracycline resistance is mediated by alternative genetic mechanisms not screened in this study (e.g., mutations in topoisomerase IV genes or efflux pump overexpression, and ribosomal protection proteins other than *TetM*). Isolate MDR‐05 (FOX–AMP–CIP–ERY–GENT–AMC–SXT) harbored *mecA*, *gyrA*, and *gyrB*, consistent with cefoxitin and ciprofloxacin resistance. Among the eight isolates (MDR‐06 to MDR‐12) with the FOX–AMP–CIP–GENT–AMC–SXT phenotype, all harbored *mecA*, *gyrA*, and *gyrB*, consistent with their cefoxitin and ciprofloxacin resistance. A single exception (MDR‐13) lacked *mecA* despite displaying cefoxitin resistance, carrying only *gyrB*, suggesting that alternative mechanisms such as β‐lactamase hyperproduction may underlie the FOX resistance phenotype in this isolate. Isolate MDR‐14 (FOX–AMP–CIP–ERY–GENT–AMC) harbored *mecA*, *gyrA*, and *gyrB*, consistent with its β‐lactam and fluoroquinolone resistance. Isolate MDR‐15 (FOX–AMP–CIP–GENT–AMC) harbored *mecA* but lacked detectable *gyrA* and *gyrB*, indicating that its ciprofloxacin resistance is likely mediated by alternative mechanisms, such as mutations in topoisomerase IV genes (*parC* or *parE*) or active efflux pump expression, not screened here. Isolates MDR‐16 (AMP–TET–SXT) and MDR‐17 (ERY–TET–SXT) harbored *tetM* as the sole detected genetic determinant, consistent with their tetracycline resistance phenotype. Across all 17 molecularly characterized MDR isolates (two MDR isolates—FOX–AMP–SXT and AMP–ERY–SXT—could not be screened due to insufficient DNA yield), resistance to gentamicin (GENT), erythromycin (ERY), amoxicillin–clavulanic acid (AMC), and trimethoprim–sulfamethoxazole (SXT) could not be attributed to the genes screened in this study, indicating the involvement of additional resistance mechanisms—such as aminoglycoside‐modifying enzymes, *erm* genes, or *dfr*/*sul* genes—that were not screened in this study.

**TABLE 6:** Individual‐level genotypic profiles of molecularly screened MDR *S. aureus* isolates.

Isolate ID	MDR phenotype	*mecA*	*mecC*	*gyrA*	*gyrB*	*tetM*
MDR‐01	FOX–AMP–CIP–ERY–GENT–TET–AMC–SXT	+	−	+	+	+
MDR‐02	FOX–AMP–CIP–ERY–GENT–TET–AMC–SXT	+	−	+	+	+
MDR‐03	FOX–AMP–CIP–ERY–GENT–TET–AMC–SXT	+	−	+	+	+
MDR‐04	FOX–AMP–CIP–ERY–GENT–TET–AMC–SXT	+	−	−	−	−
MDR‐05	FOX–AMP–CIP–ERY–GENT–AMC–SXT	+	−	+	+	−
MDR‐06	FOX–AMP–CIP–GENT–AMC–SXT	+	−	+	+	−
MDR‐07	FOX‐AMP‐CIP‐GENT‐AMC‐SXT	+	−	+	+	−
MDR‐08	FOX–AMP–CIP–GENT–AMC–SXT	+	−	+	+	−
MDR‐09	FOX–AMP–CIP–GENT–AMC–SXT	+	−	+	+	−
MDR‐10	FOX–AMP–CIP–GENT–AMC–SXT	+	−	+	+	−
MDR‐11	FOX–AMP–CIP–GENT–AMC–SXT	+	−	+	+	−
MDR‐12	FOX–AMP–CIP–GENT–AMC–SXT	+	−	+	+	−
MDR‐13	FOX–AMP–CIP–GENT–AMC–SXT	−	−	−	+	−
MDR‐14	FOX–AMP–CIP–ERY–GENT–AMC	+	−	+	+	−
MDR‐15	FOX–AMP–CIP–GENT–AMC^a^	+	−	−	−	−
MDR‐16	AMP–TET–SXT	−	−	−	−	+
MDR‐17	ERY–TET–SXT	−	−	−	−	+

*Note:* Key: FOX (cefoxitin), AMP (ampicillin), CIP (ciprofloxacin), ERY (erythromycin), GENT (gentamicin), TET (tetracycline), AMC (amoxicillin–clavulanic acid), SXT (sulfamethoxazole–trimethoprim). +, gene detected; −, gene not detected.

^a^MDR‐15 (phenotype FOX–AMP–CIP–GENT–AMC) harbored *mecA* without detectable *gyrA* or *gyrB*, suggesting that alternative mechanisms mediate its ciprofloxacin resistance. Two MDR isolates (FOX–AMP–SXT and AMP–ERY–SXT) could not be molecularly characterized due to insufficient DNA yield and are therefore not represented in this table.

### 3.6. Sequence Analysis of Selected *S. aureus* Isolates

BLASTn sequence analysis revealed that all 11 selected MRSA isolates carried the *mec*A resistance gene, with sequences showing strong homology to *S. aureus* (Table 7). Among the isolates, 469R, 487R, and 493R demonstrated the highest similarity, each showing 99% nucleotide identity (Table 7). A slightly lower identity of 98% was observed in isolates 471R, 476R, 491R, and 494F, while 484R and 479R aligned at 97% and 96%, respectively (Table 7). The lowest identities were recorded for isolates 80R (91%) and 115R (90%) (Table 7).

**TABLE 7:** MRSA genes from selected isolates, their sequenced homologue, and identity obtained from NCBI GenBank using nucleotide–nucleotide BLASTn.

Isolate ID	Target gene	Homologue	*E*‐value	Nucleotide % identity	Accession number
80R	*mec*A gene	*S. aureus*	4*e* − 18	91	
115R	*mec*A gene	*S. aureus*	3*e* − 138	90	
469R	*mec*A gene	*S. aureus*	0	99	OL597598
470R	*mec*A gene	*S. aureus*	9*e* − 64	98	OL597600
471R	*mec*A gene	*S. aureus*	0	98	OL597599
476R	*mec*A gene	*S. aureus*	0	98	OL597601
479R	*mec*A gene	*S. aureus*	5*e* − 127	96	OL597602
484R	*mec*A gene	*S. aureus*	0	97	OL597604
487R	*mec*A gene	*S. aureus*	0	99	OL597605
491R	*mec*A gene	*S. aureus*	0	98	OL597606
493R	*mec*A gene	*S. aureus*	5*e* − 137	99	OL597603

In terms of alignment quality, isolates 469R, 471R, 476R, 484R, 487R, 491R, and 494F exhibited complete alignments with no nucleotide gaps and highly significant *E*‐values of 0 (Table 7). By contrast, isolates 80R and 115R showed alignment gaps of 3% and 2%, respectively, though their *E*‐values still indicated statistically robust matches (Table 7).

## 4. Discussion

*Staphylococcus aureus* is the leading cause of zoonotic disease and the potential transmitter of antibiotic resistance strains, including MRSA, between humans and livestock. This is mediated by close interaction between domesticated animals and humans and consumption of livestock products, especially milk, and meat, which may be contaminated with staphylococcal enterotoxins [6, 29]. While *S. aureus* antibiotic resistance is a de novo phenomenon, it is frequently accelerated by the bacterium’s adaption to indiscriminate antimicrobial use in humans and animals and the haphazard use of disinfectants in farms and households [30]. Therefore, characterizing *S. aureus* isolated from human clinical blood phenotypically and genotypically is significant in determining antimicrobial‐resistant phenotypes and predominant strains, which can help curb and control the dissemination of *S. aureus* and its variant MRSA in the healthcare systems.

In this study, the prevalence of *S. aureus* in human blood samples was high, similar to a recent survey in Kenya [29], but was slightly lower compared to earlier studies in Kenya and Uganda [31, 32]. Moreover, these findings corroborate previous reports from Gabon and Côte de’Ivoire [33], which indicated higher prevalence than those reported in three in‐patient hospitals in Kenya [34, 35]. Overall, we observed that *S. aureus* is common and is frequently detected in human clinical blood in Kenya, and new methods of controlling its occurrence must be developed and implemented to avert the rising trends.

The resistance to various antimicrobials, such as ampicillin, cefoxitin, amoxicillin/clavulanic acid, sulfamethoxazole/trimethoprim, and tetracycline in this study may be due to their frequent use in healthcare practice to treat bacterial and protozoal infections in Kenya [36]. Moreover, selective pressures exerted upon *S. aureus* among other clinically significant microbes, which lead to β‐lactam antibiotic resistance, may explain the high resistance rates reported in our study. Notably, the observed high resistance to sulfamethoxazole–trimethoprim conforms to the findings of a previous report from Kenya [37] but is higher than the rates reported in Côte d’Ivoire and [33] another study in Kenya [34], Iran [24], Ethiopia [38], and Turkey [39]. In Kenya, trimethoprim/sulfamethoxazole is used as a prophylactic antimicrobial agent among patients with HIV/AIDs to prevent *Pneumocystis carinii*, which causes HIV/AID‐associated pneumonia in patients. Therefore, the selective pressure exerted by the frequent use may be linked to the emergence of *S. aureus* resistance [40]. Additionally, the high resistance of *S. aureus* to trimethoprim/sulfamethoxazole may be due to using other sulfonamides, such as sulfamethoxypyridazine/pyrimethamine and pyrimethamine/sulfadoxine, to treat malaria patients [40].

The emergence of MDR *S. aureus*, especially MRSA, poses a significant public health concern, characterized by high morbidity and mortality globally [41]. For instance, MDR coagulase–positive *S. aureus* (MRSA) and their associated enterotoxins present a significant public health concern [42]. The potential contamination of dairy products, such as raw milk, meat, among other products, with MRSA strains harboring enterotoxin genes underscores the importance of comprehensive surveillance and mitigation strategies in the food chain [43]. In regions like Kenya, where informal settlements often lack robust food safety infrastructure, the risk of transmission of MRSA strains and their enterotoxin genes through animal‐origin foodstuffs is heightened [44]. Consequently, there is a pressing need for targeted interventions aimed at enhancing food safety protocols and promoting antimicrobial stewardship in livestock farming, dairy production, and healthcare sectors [45].

The emergence and rapid spread of MDR *S. aureus* strains worldwide complicate the management of staphylococcal infection [44]. The multiple resistance phenotypes among the *S. aureus* isolates obtained from the clinical isolates may be due to the acquisition of plasmid‐mediated resistance factors (R‐factors) as described in a related previous study [46]. MDR is clinically defined as resistance to three or more classes of antibiotics [23]. In the present study, 35.85% of the *S. aureus* isolates were MDR. This finding concurs with a report from ABUTH, Zaria [47]; however, the observed frequency was lower than that reported in Nepal (44.2%) [48] and Northeast Ohio, USA (92.9%) [49].

In this study, the MDR phenotypes FOX–AMP–CIP–GENT–AMC–SXT and FOX–AMP–CIP–ERY–GENT–TET–AMC–SXT were predominantly observed. Previous studies have shown that the emergence and spread of MDR *S. aureus* are often driven by inappropriate antimicrobial use in both veterinary and human medicines, including self‐prescription and unregulated access to antibiotics [36]. The high frequency of MDR *S. aureus* observed in our study may similarly reflect indiscriminate antibiotic use and limited awareness of antimicrobial resistance among residents of Mukuru Slum, where the study was conducted. Besides, the phenotypic resistance patterns corresponded with the detection of key genetic determinants. Specifically, the cefoxitin (FOX) and ampicillin (AMP) resistance profiles were associated with carriage of the *mec*A gene, a well‐established determinant of methicillin resistance [4]. Resistance to ciprofloxacin (CIP) and gentamicin (GENT) correlated with mutations in *gyr*A and *gyr*B, which mediate alterations in DNA *gyrA*se targeted by fluoroquinolones [50], while tetracycline (TET) resistance was explained by the presence of the *tet*M gene [51]. Amoxicillin–clavulanic acid (AMC) and sulfamethoxazole–trimethoprim (SXT) resistance were phenotypically observed but could not be conclusively linked to the genes analyzed in this study, suggesting the possible contribution of other mechanisms not screened here.

Overall, 82.35% of MDR isolates were MRSA, with the majority harboring *mecA* in addition to *gyr*A*, gyr*B, and *tet*M. These findings indicate that *mec*A was the predominant genetic determinant underpinning methicillin‐resistant phenotypes, while *gyr*A/B and *tet*M accounted for fluoroquinolone and tetracycline resistance, respectively. This genotype–phenotype concordance is consistent with previous reports from pastoral regions [36] and canine surgical wounds in Kenya [52]. Nonetheless, the absence of *mecA* in some phenotypically resistant isolates has also been reported globally [53]. Such discordance may be attributable to alternative resistance mechanisms, including hyperproduction of β‐lactamases [54] or the presence of other methicillin resistance genes such as *mecB* and *mecC* [53].

The distribution of MRSA can be determined by analyzing the genes conserved within the staphylococcal cassette chromosome (*SCCmec*) encoding mutant PBP2a or PBP2 [55]. PBP2a protein decreases affinity to methicillin and most beta‐lactam antibiotics, including carbapenems except for ceftaroline and ceftobiprole [6]. Besides, resistance among MRSA isolates depends on the ability of *S. aureus* to produce PBP2a, which can be influenced by several chromosomal and extra‐chromosomal factors [54]. Overall, the *mecA* gene was detected in 85% of *S. aureus* isolates obtained from blood samples from patients seeking outpatient services at healthcare facilities in the Mukuru slum, and none detected the *mecC* gene by PCR assay, depicting the diverse and dynamic nature of AMR and MDR resistance [56].

Efforts to combat the transmission of MRSA and its enterotoxin genes must encompass a multidisciplinary approach, integrating veterinary medicine, public health, and food safety expertise, as well as implementing stringent surveillance measures along the production, processing, and distribution stages of animal‐derived food products is essential to identify and mitigate potential sources of contamination [45]. Moreover, education and outreach programs are instrumental in raising awareness about the risks associated with MRSA in foodstuffs and healthcare setting and fostering adherence to best practices for food safety and antimicrobial use. By addressing these challenges comprehensively, stakeholders can mitigate the public health risks posed by MRSA and assure public health and safety.

## 5. Limitations

This study was conducted in outpatient health facilities located in Mukuru Slum, an informal urban settlement in Nairobi, Kenya. The setting is characterized by high population density, inadequate sanitation, and limited healthcare access. These socioenvironmental conditions are likely to contribute to the circulation of antimicrobial‐resistant *S. aureus*, including MRSA. While these contextual factors strengthen the public health relevance of our findings, they also mean the results should be interpreted cautiously and not directly generalized to regions with different socioeconomic and healthcare profiles.

In addition, the study design relied on convenience sampling of patient presenting during the study period, and demographic or clinical characteristics of participants (such as age, sex, hospitalization history, or prior antibiotic use) were not collected. This limitation reduced our ability to assess individual‐level risk factors. Sequencing was confined to a selected subset of *nuc* and *mec*A amplicons rather than comprehensive genomic analysis, which constrained insight into strain diversity and clonal relationships. Thus, future work should assess fluoroquinolone resistance determinants across all ciprofloxacin‐resistant isolates regardless of methicillin resistance status. Despite these limitations, the study provides important baseline data from a neglected high‐burden setting and highlights the need for strengthened surveillance and antimicrobial stewardship in similar environments.

## 6. Conclusion and Recommendations

This study confirms that *Staphylococcus aureus*, including MRSA, is a significant cause of bloodstream infections in outpatient facilities in Mukuru Slum, Nairobi. More than one‐third of isolates exhibited MDR, with phenotypic patterns largely explained by carriage of *mecA, gyrA, gyrB,* and *tetM* genes. Sequencing of 10 selected *nuc* and *mecA* amplicons confirmed the molecular identification of *S. aureus* and validated the accuracy of genotypic detection. The observed MDR in over one‐third of isolates (35.85%) corresponded closely with the presence of resistance genes (*mecA*, *gyrA*, *gyrB*, and *tetM*), indicating a strong relationship between phenotypic resistance patterns and underlying genetic determinants. Although ciprofloxacin, erythromycin, gentamicin, and amoxicillin–clavulanic acid showed comparatively lower resistance rates, the high overall prevalence of AMR and MDR underscores the urgent need for enhanced antimicrobial resistance surveillance in this high‐burden setting.

To mitigate the risks posed by MDR *S. aureus* and MRSA, targeted strategies including strengthening routine antimicrobial resistance surveillance, screening and managing potential carriers to interrupt transmission, and implementing antimicrobial stewardship programs tailored to resource‐limited environments are recommended. Community‐level interventions addressing sanitation, infection prevention, and responsible antibiotic use are equally critical. Future research incorporating whole‐genome sequencing would provide deeper insights into clonal diversity and transmission dynamics, supporting more effective control policies in similar socioenvironmental contexts.

## Author Contributions

Jeremiah Ogeto, Simon Mitema, and Gabriel Aboge conceptualized and designed the study; Simon Mitema, Gabriel Aboge, Gervason Moriasi, and Alfred Mainga oversaw the design and collection of samples; Jeremiah Ogeto, Alfred Mainga, and Gabriel Aboge performed and supervised the experiments; Gabriel Aboge, Simon Mitema, Gervason Moriasi, and Jeremiah Ogeto performed data analysis including sequence analysis; and Jeremiah Ogeto, Simon Mitema, Gervason Moriasi, and Gabriel Aboge wrote the manuscript.

## Funding

This study did not receive any formal funding.

## Disclosure

Part of the results reported in this research article was previously published, which was submitted to the University of Nairobi [46]. All authors reviewed the manuscript and approved the final draft for submission and publication.

## Conflicts of Interest

The authors declare no conflicts of interest.

## Supporting Information

Supporting Figures (SF. 1A‐ H): Culture and biochemical characteristics for the identification of *S. aureus* in the samples studied.

Supporting Figure (SF. 2): Ethidium bromide–stained 1.5% w/v agarose gel electrophoresis of the *nuc* gene in *S. aureus* isolates.

Supporting Table 1 (ST.1): *S. aureus* isolates recovered on culture and confirmed as positive isolates of *S. aureus* using biochemical tests.

Supporting Table 2 (ST. 2): Antimicrobial susceptibility testing for *S. aureus* isolates from human blood based on the disk diffusion technique.

## Supporting information


**Supporting Information** Additional supporting information can be found online in the Supporting Information section.

## Data Availability

All data are included in the manuscript and its supporting files; however, additional information may be provided upon reasonable request.
