# Supplementary material for: Phenotypic and Genotypic Analyses of Antimicrobial Resistance Patterns of Staphylococcus aureus Isolates From Outpatient Blood Samples in Mukuru Slum, Nairobi, Kenya
Source: Can J Infect Dis Med Microbiol. 2026 Apr 28;2026:3974296. doi: 10.1155/cjid/3974296 (PMC13125862; doi:10.1155/cjid/3974296)
Supplement: Supplementary file 1 — Supporting Information Additional supporting information can be found online in the Supporting Information section. [file CJID-2026-3974296-s001.pdf]

Supplementary results

Culture and biochemical characteristics of human blood samples

Out of 142 human blood samples that were cultured on Mannitol Salt Agar (MSA), 140 (98.6%) showed a characteristic small, circular, smooth yellow colony on MSA with a colour change on media from phenol red to yellow conforming to *S. aureus* identity (**SF.1 A and B**). All the 140 *S. aureus* isolates were then cultured on Sheep Blood Agar (SBA) and 112 (78.9%) showed a clear zone around the colonies, indicating a positive β-haemolytic effect of *S. aureus* on SBA (**SF. 1C and D**). All the 112 (100%) isolates showed an active bubbling on reaction with 3% v/v hydrogen peroxide on catalase test, indicating a positive presumptive isolates of *S. aureus* (**SF. 1G**). Out of 112 isolates from human blood, 56 (39.4%) showed a phenomenal clotting with varied grades of coagulation hence a positive coagulase test indicating isolates of *S. aureus* (**SF. 1E, F, and H)**. Observations from culture and biochemical test shows that 56 (39.4%) isolates were confirmed as positive *S. aureus* (**ST. 1**).

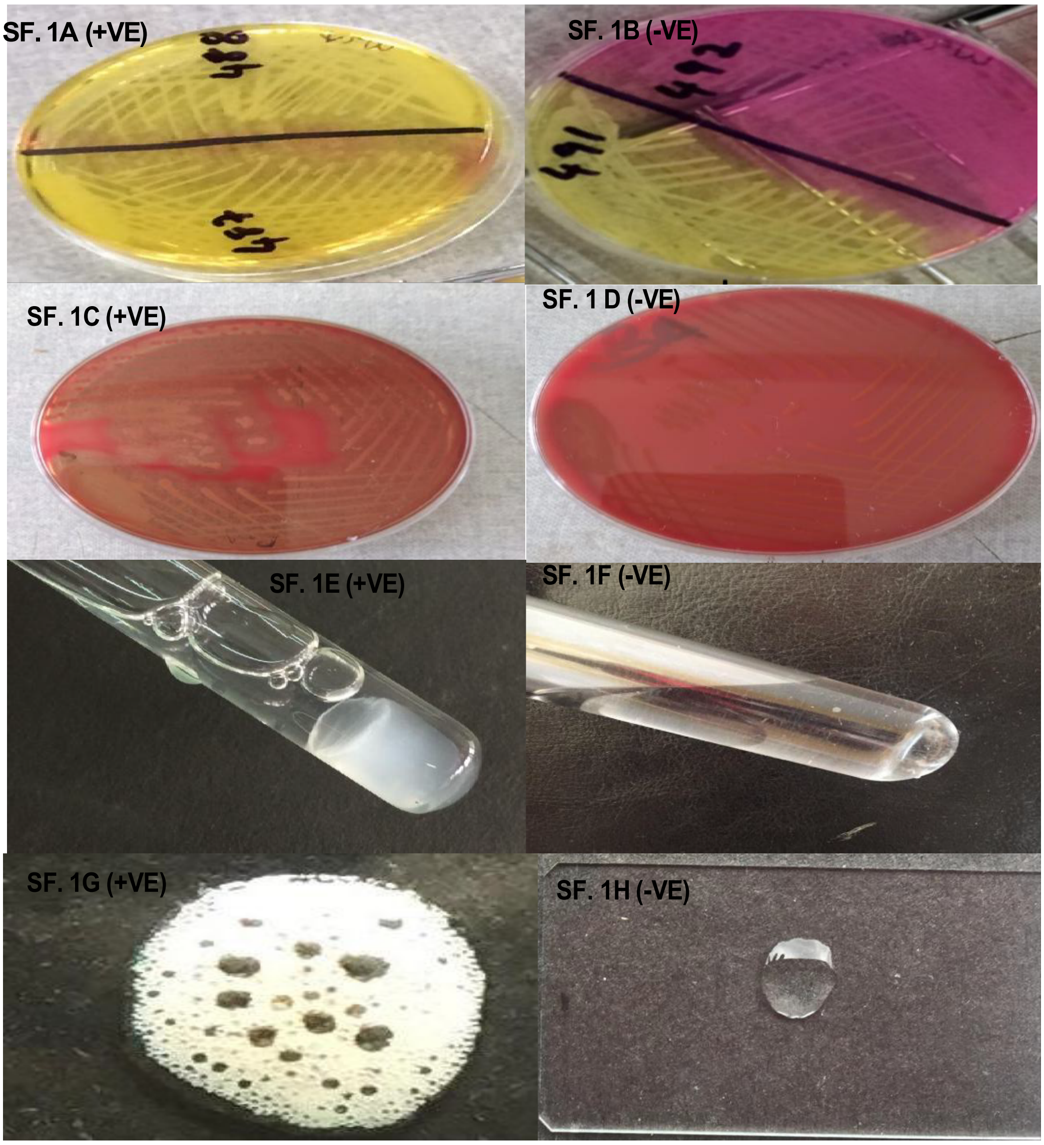

SF. 1A- H: Culture and biochemical characteristics for the identification of *S. aureus* in the studied samples.

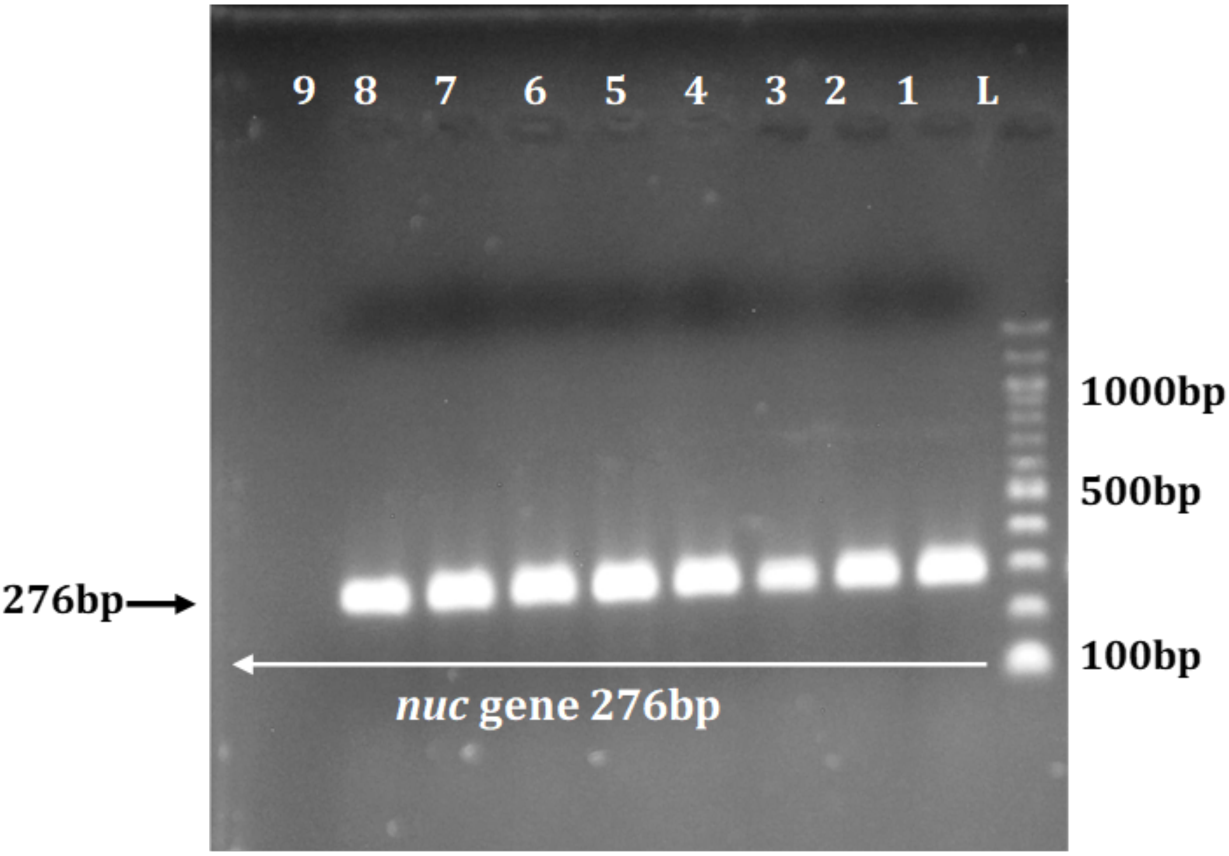

SF. 2: Ethidium bromide-stained 1.5% w/v agarose gel electrophoresis of the *nuc* gene in *S. aureus* isolates.
From right to Left, Lane 1- positive control (*S. aureus*, ATCC 25923); Lane 9- negative control (purified water); Positive *S. aureus* in Lane 2 to 8 and L is a 100-bp molecular ladder.

ST 1: *S. aureus* isolates recovered on culture and confirmed as positive isolates of *S. aureus* using biochemical tests

samples						
**Sample Source**	**No. of Samples (N)**	**Growth on MSA (n%)**	**β-Haemolysis**	**Growth on TSA (n (%)**	**Catalase test (n%)**	**Coagulase test (n%)**
Human (Blood)	142	140(98.6%)	112(78.9%)	112(100%)	112(100%)	56(39.4%)

MSA-Mannitol Salt Agar, TSA- Tryptic Soy Agar.

ST. 2: Antimicrobial susceptibility testing for S. aureus isolates from human blood based on the disk diffusion technique

Sample No.	Diameter (mm)	FOX 30µg	AMP 10µg	CIP 5µg	ERY 15µg	GENT 10µg	TET 30µg	AMC 30µg	SXT 25µg
ATCC 25923	D1	28	28	25	25	28	27	25	29
	D2	30	24	26	23	28	23	25	29
	AVERAGE	29	26	26	24	28	25	25	29
3	D1	28	27	26	24	19	8	25	30
	D2	28	25	26	24	19	9	25	30
	AVERAGE	28	26	26	27	19	9	25	30
4	D1	28	16	23	28	20	8	22	24
	D2	27	16	23	26	20	8	22	24
	AVERAGE	28	16	23	27	20	8	22	24
8	D1	28	18	28	25	19	9	24	28
	D2	28	18	26	25	19	9	24	26
	AVERAGE	28	18	27	25	19	9	24	27
10	D1	28	20	30	27	20	8	30	24
	D2	28	20	30	25	20	9	30	24
	AVERAGE	28	20	30	26	20	9	30	24
11	D1	30	18	25	27	21	11	25	22
	D2	30	18	25	25	19	11	25	22
	AVERAGE	30	18	25	26	20	11	25	22
12	D1	30	20	24	30	23	12	24	20
	D2	28	18	24	30	23	10	24	21
	AVERAGE	29	19	24	30	23	11	24	21
13	D1	30	18	24	29	20	9	28	6
	D2	29	19	25	28	20	9	29	6
	AVERAGE	30	19	25	29	20	9	29	6
14	D1	30	22	28	30	22	29	30	6
	D2	30	23	29	30	21	28	30	6
	AVERAGE	30	23	29	30	22	29	30	6
15	D1	32	18	28	6	23	30	26	6
	D2	31	16	27	6	22	28	25	6
	AVERAGE	32	17	28	6	23	29	26	6
16	D1	30	21	32	28	22	29	30	6
	D2	29	20	32	28	21	28	30	6
	AVERAGE	30	21	32	28	22	29	30	6
18	D1	30	20	28	6	22	10	30	6
	D2	29	20	26	6	21	10	30	6
	AVERAGE	30	20	27	6	22	10	30	6
19	D1	29	20	29	30	21	6	27	18
	D2	30	21	28	29	21	6	26	17

Sample No.	Diameter (mm)	FOX 30µg	AMP 10µg	CIP 5µg	ERY 15µg	GENT 10µg	TET 30µg	AMC 30µg	SXT 25µg
	AVERAGE	30	21	29	30	21	6	27	18
22	D1	31	21	30	29	22	30	30	6
	D2	30	21	31	28	21	31	30	6
	AVERAGE	31	21	31	29	22	31	30	6
24	D1	30	19	30	29	20	30	31	8
	D2	31	18	31	30	21	31	30	9
	AVERAGE	31	19	31	30	21	31	31	9
25	D1	35	19	28	30	25	30	25	26
	D2	33	17	27	31	25	29	25	25
	AVERAGE	34	18	28	31	25	30	25	26
27	D1	29	20	30	27	22	9	29	23
	D2	28	21	31	26	21	8	30	24
	AVERAGE	29	21	31	27	22	9	30	24
28	D1	32	22	24	30	24	33	33	6
	D2	31	22	25	30	23	32	31	6
	AVERAGE	32	22	25	30	24	33	32	6
29	D1	30	24	27	30	24	10	25	25
	D2	30	22	26	30	23	10	26	24
	AVERAGE	30	23	27	30	24	10	26	25
30	D1	30	20	29	30	21	9	29	6
	D2	29	20	28	30	20	9	28	6
	AVERAGE	30	20	29	30	21	9	29	6
31	D1	29	18	26	25	22	9	24	6
	D2	28	19	25	26	22	9	24	6
	AVERAGE	29	19	26	26	22	9	24	6
32	D1	30	19	28	30	23	30	27	6
	D2	29	17	28	32	25	32	26	6
	AVERAGE	30	18	28	31	24	31	27	6
33	D1	27	18	26	28	18	28	22	24
	D2	25	18	26	27	19	26	23	24
	AVERAGE	26	18	26	28	19	27	23	24
34	D1	28	14	27	6	20	30	23	6
	D2	28	12	26	6	21	29	23	6
	AVERAGE	28	13	27	6	21	30	23	6
35	D1	25	17	28	6	19	28	26	6
	D2	25	17	27	6	18	27	25	6
	AVERAGE	25	17	28	6	19	28	26	6
36	D1	29	15	25	30	20	30	23	22
	D2	28	14	26	29	21	29	23	21
	AVERAGE	29	15	26	30	21	30	23	22

Sample No.	Diameter (mm)	FOX 30µg	AMP 10µg	CIP 5µg	ERY 15µg	GENT 10µg	TET 30µg	AMC 30µg	SXT 25µg
37	D1	30	15	28	30	18	10	22	23
	D2	29	16	28	29	18	9	22	24
	AVERAGE	30	16	28	30	18	10	22	24
38	D1	29	16	23	28	19	29	22	24
	D2	28	16	22	27	19	28	22	24
	AVERAGE	29	16	23	28	19	29	22	24
39	D1	25	13	27	24	18	8	23	6
	D2	25	14	26	25	17	8	24	6
	AVERAGE	25	14	27	25	18	8	24	6
78	D1	26	20	28	28	18	29	26	6
	D2	26	21	26	29	18	29	26	6
	AVERAGE	26	21	27	29	18	29	26	6
79	D1	28	13	28	26	18	28	22	11
	D2	29	12	27	27	19	29	21	10
	AVERAGE	29	13	28	27	19	29	22	11
80	D1	21	13	30	28	19	30	23	24
	D2	21	12	29	29	19	29	22	22
	AVERAGE	21	13	30	29	19	30	23	23
92	D1	26	13	26	27	19	26	22	10
	D2	16	14	26	26	19	25	23	9
	AVERAGE	21	14	26	27	19	26	23	10
93	D1	21	20	30	30	22	30	26	25
	D2	21	20	29	30	23	29	25	24
	AVERAGE	21	20	30	30	23	30	26	25
103	D1	23	18	29	27	20	28	24	20
	D2	19	18	29	27	19	29	24	19
	AVERAGE	21	18	29	27	20	29	24	20
104	D1	26	16	27	28	16	29	22	22
	D2	26	14	26	28	12	27	22	22
	AVERAGE	26	15	27	28	14	28	22	22
115	D1	14	6	6	6	12	26	12	6
	D2	15	6	6	6	12	27	12	6
	AVERAGE	15	6	6	6	12	27	12	6
469	D1	14	6	6	6	12	6	11	6
	D2	15	6	6	6	11	6	12	6
	AVERAGE	15	6	6	6	12	6	12	6
470	D1	11	6	12	17	10	24	10	6
	D2	10	6	12	18	9	23	9	6
	AVERAGE	11	6	12	18	10	24	10	6
471	D1	16	6	6	20	11	25	12	6

Sample No.	Diameter (mm)	FOX 30µg	AMP 10µg	CIP 5µg	ERY 15µg	GENT 10µg	TET 30µg	AMC 30µg	SXT 25µg
	D2	15	6	6	19	10	24	13	6
	AVERAGE	16	6	6	20	11	25	13	6
475	D1	10	6	6	6	7	7	12	6
	D2	9	6	6	6	6	8	11	6
	AVERAGE	10	6	6	6	7	8	12	6
476	D1	11	7	6	13	10	23	14	6
	D2	12	8	6	14	9	22	13	6
	AVERAGE	12	8	6	14	10	23	14	6
479	D1	10	6	10	11	9	21	15	15
	D2	12	7	11	10	8	20	14	16
	AVERAGE	11	7	11	11	9	21	15	16
482	D1	12	6	6	14	10	22	10	6
	D2	11	6	6	13	9	23	11	6
	AVERAGE	12	6	6	14	10	23	11	6
484	D1	11	6	6	16	10	25	12	6
	D2	12	6	6	15	11	25	13	6
	AVERAGE	12	6	6	16	11	25	13	6
485	D1	9	6	8	15	9	23	12	14
	D2	10	6	9	16	10	23	11	16
	AVERAGE	10	6	9	16	10	23	12	15
486	D1	25	14	25	25	21	26	30	25
	D2	26	14	25	25	21	25	31	26
	AVERAGE	26	14	25	25	21	26	31	26
487	D1	7	6	10	14	10	20	10	6
	D2	9	6	9	14	9	21	10	6
	AVERAGE	8	6	10	14	10	21	10	6
488	D1	10	6	6	6	10	7	12	6
	D2	11	6	6	6	11	8	11	6
	AVERAGE	11	6	6	6	11	8	12	6
490	D1	12	6	7	16	9	23	11	6
	D2	11	6	6	16	10	22	12	6
	AVERAGE	12	6	7	16	10	23	12	6
491	D1	14	10	26	26	19	25	20	28
	D2	15	11	22	25	20	24	21	27
	AVERAGE	15	11	24	26	20	25	21	28
493	D1	13	7	7	6	12	6	11	6
	D2	12	6	6	6	12	6	12	6
	AVERAGE	13	7	7	6	12	6	12	6
494	D1	12	6	6	16	10	24	12	6
	D2	11	6	6	15	11	24	11	6

Sample No.	Diameter (mm)	FOX 30µg	AMP 10µg	CIP 5µg	ERY 15µg	GENT 10µg	TET 30µg	AMC 30µg	SXT 25µg
	AVERAGE	12	6	6	16	11	24	12	6
495	D1	26	18	28	29	24	30	25	33
	D2	27	19	27	30	24	31	26	32
	AVERAGE	27	19	28	30	24	31	26	33

KEY: **Resistant**

Intermediate Resistant

Susceptible
